# BKIP-1, an auxiliary subunit critical to SLO-1 function, inhibits SLO-2 potassium channel *in vivo*

**DOI:** 10.1038/s41598-017-18052-z

**Published:** 2017-12-19

**Authors:** Long-Gang Niu, Ping Liu, Yuan Shui, Roger Mailler, Zhao-Wen Wang, Bojun Chen

**Affiliations:** 1 Department of Neuroscience, UConn Health, Farmington CT, USA; 2 Department of Computer Science, University of Tulsa, Tulsa, OK USA

## Abstract

Auxiliary subunits are often needed to tailor K^+^ channel functional properties and expression levels. Many auxiliary subunits have been identified for mammalian Slo1, a high-conductance K^+^ channel gated by voltage and Ca^2+^. Experiments with heterologous expression systems show that some of the identified Slo1 auxiliary subunits can also regulate other Slo K^+^ channels. However, it is unclear whether a single auxiliary subunit may regulate more than one Slo channel in native tissues. BKIP-1, an auxiliary subunit of *C. elegans* SLO-1, facilitates SLO-1 membrane trafficking and regulates SLO-1 function in neurons and muscle cells. Here we show that BKIP-1 also serves as an auxiliary subunit of *C. elegans* SLO-2, a high-conductance K^+^ channel gated by membrane voltage and cytosolic Cl^−^ and Ca^2+^. Comparisons of whole-cell and single-channel SLO-2 currents in native neurons and muscle cells between worm strains with and without BKIP-1 suggest that BKIP-1 reduces chloride sensitivity, activation rate, and single-channel open probability of SLO-2. Bimolecular fluorescence complementation assays indicate that BKIP-1 interacts with SLO-2 carboxyl terminal. Thus, BKIP-1 may serve as an auxiliary subunit of SLO-2. BKIP-1 appears to be the first example that a single auxiliary subunit exerts opposite effects on evolutionarily related channels in the same cells.

## Introduction

The Slo family of K^+^ channels in mammals include Slo1 (BK channel), Slo2.1 (*Slick*), Slo2.2 (*Slack*), and Slo3. These channels are gated by membrane voltage and specific ions on the intracellular side (Ca^2+^ for Slo1, Ca^2+^ and Cl^−^ for Slo2, and H^+^/pH for Slo3)^1^. They are expressed in various tissues and cells, and play many important physiological roles. A variety of auxiliary/regulatory subunits have evolved to tailor the biophysical properties and expression levels of Slo channels to specific cellular needs. Two classes of auxiliary subunits have been identified for Slo1, including β subunits (β1–β4) and γ subunits (γ1–γ4)^2–12^. β and γ subunits are membrane proteins with two and one transmembrane domains, respectively. β subunits regulate Slo1 surface expression, apparent voltage and Ca^2+^ sensitivities, and rates of inactivation, activation and deactivation whereas γ subunits shift the voltage dependence of Slo1 activation toward hyperpolarizing voltages (see reviews^13–15^). In contrast to Slo1, knowledge about auxiliary/regulatory proteins for Slo2 and Slo3 is very limited. Fragile X mental retardation protein (FMRP), a RNA-binding protein, may associate with Slo2.2 and regulate its gating^16^. LRRC52/γ2 may serve as an auxiliary subunit of Slo3^17^ although it was initially identified as a regulatory protein of Slo1 based on analyses with transfected HEK293 cells^3^. Analyses with heterologous expression systems have raised the possibility that a single auxiliary subunit may regulate more than one Slo channel^3,7,12,17,18^ but it remains to be addressed whether this happens in native tissues.

The nematode *Caenorhabditis elegans (C. elegans)* has two Slo family members: SLO-1 and SLO-2, which are orthologues of mammalian Slo1 and Slo2, respectively. Both channels are expressed in neurons and muscle cells but they differ in physiological functions. In neurons, the two channels regulate neurotransmitter release through different mechanisms. SLO-1 acts at presynaptic sites, and inhibits spontaneous and evoked neurotransmitter release^19,20^ whereas SLO-2 only inhibits spontaneous neurotransmitter release^21^. In muscle cells, SLO-2 shapes action potentials^22^ whereas SLO-1 regulates Ca^2+^ homeostasis^20^. In both neurons and muscle cells, SLO-2 plays important roles in regulating cellular excitability by conducting delayed outward currents^21,22^. A recent study shows that SLO-1 and SLO-2 bidirectionally regulate ethanol withdrawal responses^23^. A variety of interacting proteins are known for SLO-1^20,24–27^ but no regulatory proteins of SLO-2 have been identified.

BKIP-1 (*BK* channel *i*nteracting *p*rotein*-1*), a single pass membrane protein, is an auxiliary subunit of SLO-1 in *C. elegans* ^24^. It elevates the half-maximal voltage for activation (*V*_50_) and slows activation rate of SLO-1 at lower [Ca^2+^] but reduces the *V*_50_ and shows no obvious effect on activation rate at higher [Ca^2+^]^24^. These effects of BKIP-1 are similar to those of β4 subunit on mammalian Slo1^6^. In addition, BKIP-1 plays an important role in SLO-1 surface expression^24^. Interestingly, a recent study shows that BKIP-1 genetically interacts with both SLO-1 and SLO-2 to control terminal differentiation of a pair of *C. elegans* olfactory neurons^28^. However, it remains to be determined how BKIP-1 may affect SLO-2 function. Here, through analyzing the effects of *bkip-1* loss-of-function mutation on SLO-2 function in *C. elegans* neurons and muscle cells, we show that BKIP-1 is an inhibitory auxiliary subunit of SLO-2. BKIP-1 causes significant decreases in SLO-2 apparent Cl^−^ and voltage sensitivities, activation rate, and single-channel open probability. These effects of BKIP-1 on SLO-2 are in contrast to those of BKIP-1 on SLO-1^24^, suggesting that a single auxiliary subunit may disparately regulate two different Slo channels within the same cells. The identification of BKIP-1 as regulators of both SLO-1 and SLO-2 reveals a new aspect of versatility of auxiliary/regulatory subunits in Slo channel functions.

## Results

### BKIP-1 reduces voltage dependence and slows activation rate of SLO-2 in body-wall muscle cells

**Figure 1:**
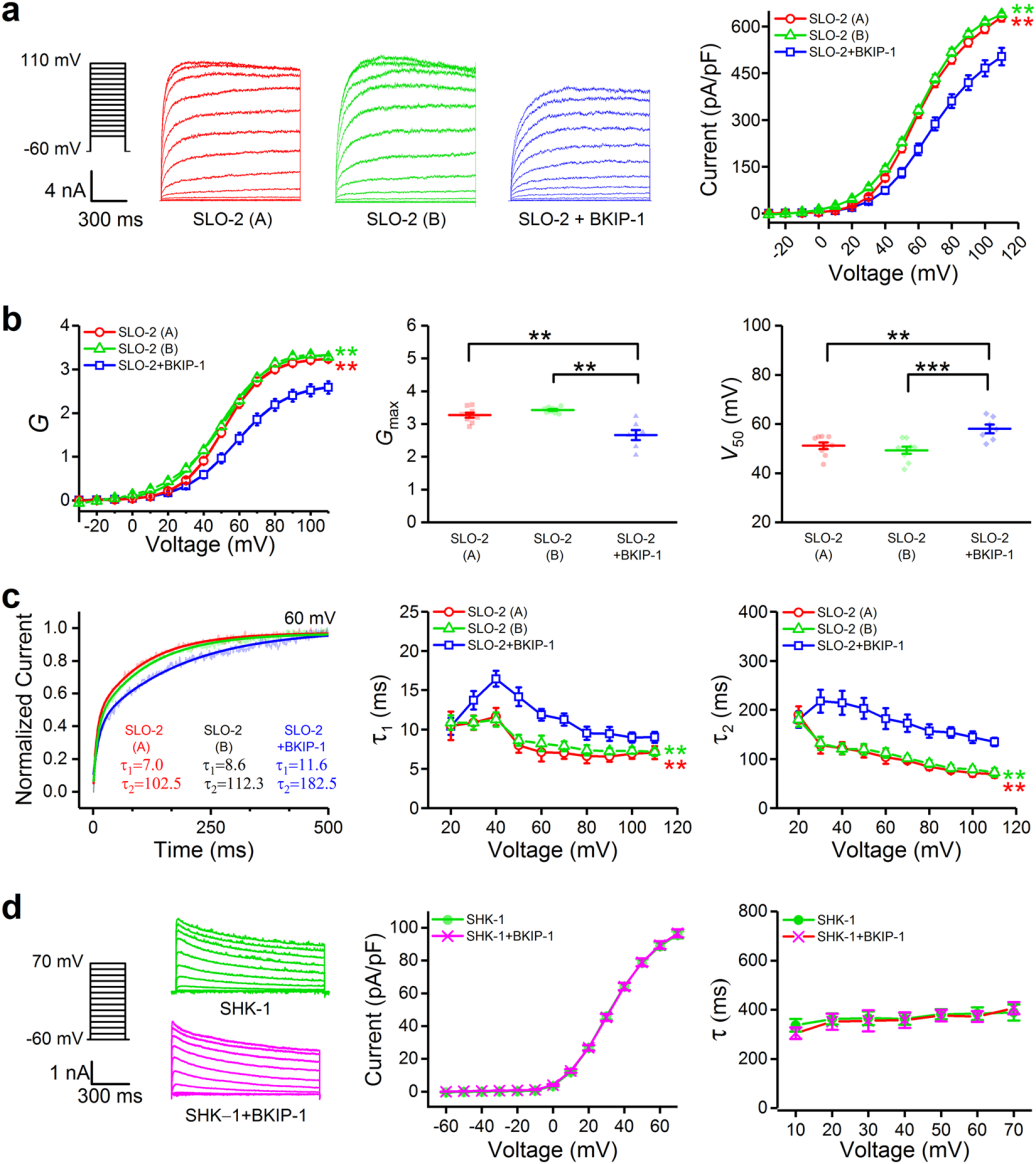
Effects of BKIP-1 on the amplitude, voltage dependence, and activation rate of SLO-2 whole-cell currents in body-wall muscle cells. (**a**) Representative current traces and current-voltage relationship of SLO-2 (A) (*n* = 9), SLO-2 (B) (*n* = 10), and SLO-2 + BKIP-1 (*n* = 7), which represent *shk-1*(*ok1581*);*bkip-1*(*zw10*), *shk-1*(*ok1581*);*bkip-1(zw17*), and *shk-1(ok1581)* strains, respectively. (**b**) Conductance (*G*) – voltage relationship of the currents fitted to a Boltzmann function, and comparison of the *G*_*max*_ and *V*_50_ from the Boltzmann fit. (**c**) Sample current traces of the initial 500 ms in response to a +60-mV voltage step fitted to two exponentials (τ_1_ and τ_2_), and comparison of the τ_1_ and τ_2_ values among the three groups. (**d**) BKIP-1 has no effect on SHK-1 whole-cell currents in body-wall muscle cells. Shown are representative current traces, current-voltage relationship, and inactivation rate of whole-cell currents from SHK-1 (*n* = 18) and SHK-1 + BKIP-1 (*n* = 21), which represent *slo-2(nf101);bkip-1(zw10)* and *slo-2(nf101)* strains, respectively. Values are shown as mean ± SE. All statistical comparisons were made with two-way ANOVA except for *G*_*max*_ and *V*_50_ in panel **b** (one-way ANOVA). The asterisks indicate statistically significant differences between SLO-2 and SLO-2 + BKIP-1 (**p < 0.01, ***p < 0.001).

BKIP-1 was initially identified as an auxiliary subunit of SLO-1^24^. During the course of that study, we tested the effect of BKIP-1 on SLO-2 using the *Xenopus* oocyte expression system with the expectation that BKIP-1 would show either no effect or an enhancing effect on SLO-2. To our surprise, we observed a strong inhibitory effect of BKIP-1 on SLO-2 macroscopic currents in inside-out patches. These observations raised the possibility that BKIP-1 is a dual regulator of SLO-1 and SLO-2 with bidirectional effects *in vivo*. To determine whether BKIP-1 regulates SLO-2 *in vivo*, we began analyses with *C. elegans* body-wall muscle cells where both proteins are expressed^24,29^. The muscle cells produce large delayed outward currents in response to depolarizing voltage steps. The delayed outward currents result from the functions of two K^+^ channels: SLO-2 and SHK-1 (a *Shaker*-type/K_V_1 K^+^ channel), which contribute approximately 80% and 20% of the currents, respectively^22^. To avoid complications by SHK-1 current, we assessed potential effects of BKIP-1 on SLO-2 function by comparing whole-cell currents between *shk-1(ok1581)* and two double mutants: *shk-1(ok1581);bkip-1(zw10)* and *shk-1(ok1581);bkip-1(zw17)*. All the *shk-1* and *bkip-1* mutants are putative nulls^22,24^. For clarity, the *shk-1*(*ok1581*)strain is henceforth referred to as SLO-2 + BKIP-1, whereas the *shk-1(ok1582);bkip-1*(*zw10*) and *shk-1(ok1581*);*bkip-1(zw17)* stains as SLO-2(A) and SLO-2(B), respectively. We first compared voltage-dependent whole-cell currents between the strains, and found that current density was significantly smaller in SLO-2 + BKIP-1 than either SLO-2(A) or SLO-2(B) (20% difference at +110 mV) (Fig. 1a). The conductance (*G*) – voltage (*V*) relationship was fitted to a Boltzmann equation to quantify the maximal conductance (*G*_*max*_) and half-maximal voltage for activation (*V*_50_) of SLO-2. BKIP-1 causes a smaller *G*_*max*_ (SLO-2(A), 3.27 ± 0.07 pS; SLO-2(B), 3.43 ± 0.03 pS; SLO-2 + BKIP-1, 2.66 ± 0.15 pS), and a rightward shift in the *G – V* relationship (*V*_50_, SLO-2(A), 51.2 ± 1.3 mV; SLO-2(B), 50.2 ± 1.1 mV; SLO-2 + BKIP-1, 58.0 ± 1.8 mV) (Fig. 1b). We then quantified SLO-2 activation rate by fitting current traces from the first 500-ms of voltage steps to two exponentials (Fig. 1c, *left*). τ_1_ and τ_2_ values of SLO-2 + BKIP-1 are significantly larger than those of either SLO-2(A) or SLO-2(B) (Fig. 1c). Taken together, BKIP-1 has several effects on SLO-2 whole-cell currents, including reducing current amplitude, decreasing apparent voltage dependence, and slowing activation rate.

To determine whether BKIP-1 also regulates SHK-1, we compared whole-cell currents between *slo-2*(*nf101*) (a deletion mutant)^30^ and *slo-2*(*nf101*);*bkip-1*(*zw10*), which are equivalent to SHK-1 + BKIP-1 and SHK-1, respectively. Both the current-voltage relationship and inactivation rate are indistinguishable between these two groups (Fig. 1d), suggesting that BKIP-1 does not regulate K^+^ channels indiscriminately.

### BKIP-1 reduces SLO-2 Cl^−^ but not Ca^2+^ sensitivity

**Figure 2:**
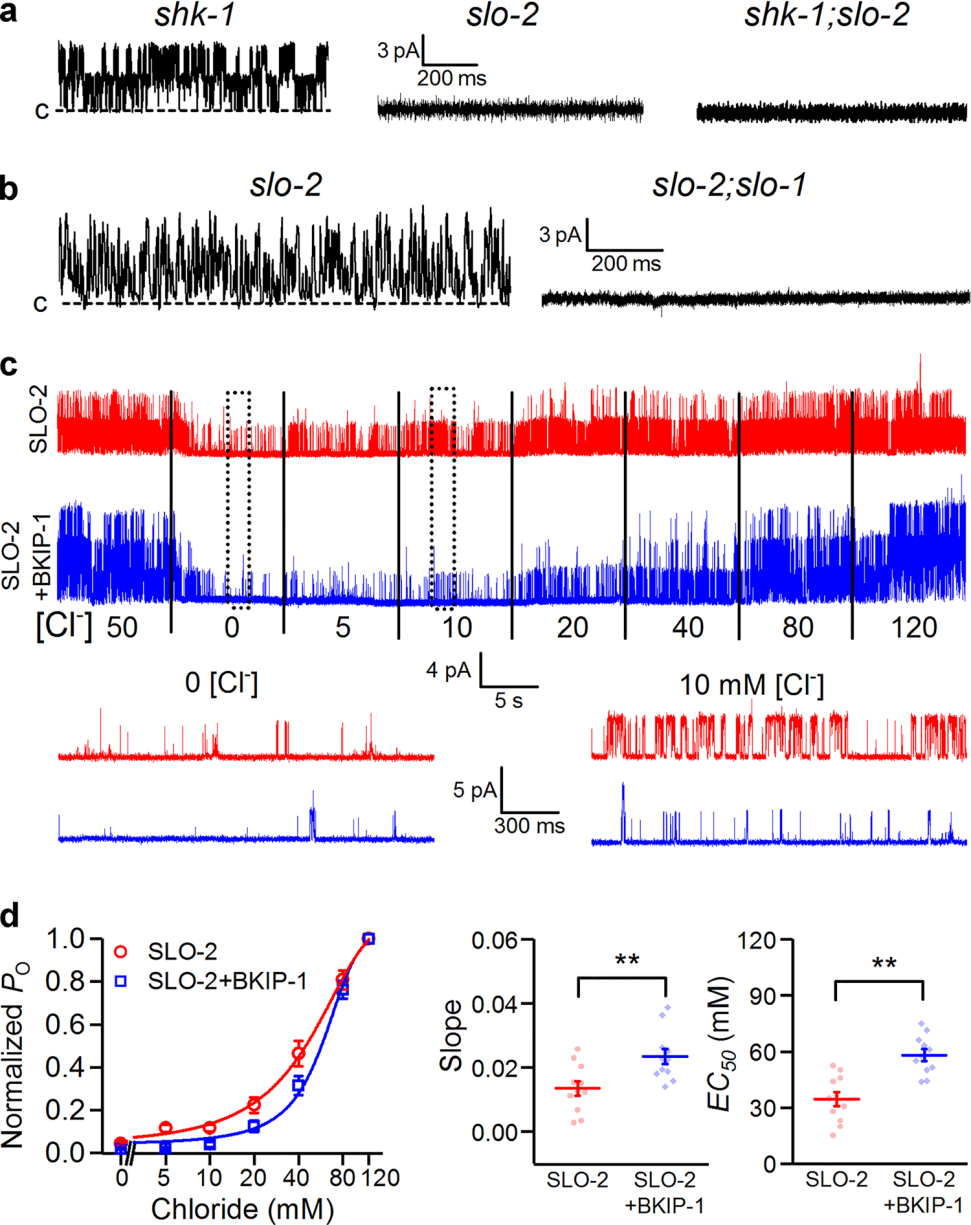
BKIP-1 reduces SLO-2 Cl^−^ sensitivity in inside-out patches of body-wall muscle cells. (**a**) SLO-2 single-channel events were observed in patches from *shk-1(ok1581)* but not from either *slo-2(nf101)* or *shk-1(ok1581);slo-2(nf101)*. The recordings were performed with pipettes of ~20 MΩ tip resistance at a holding voltage of +30 mV. (**b**) SLO-1 single channel events were observed in some patches (~20%) from *slo-2(nf101)* but not from *slo-2(nf101);slo-1(md1745*). The recordings were performed with pipettes of 2-3 MΩ tip resistance at a holding voltage of +30 mV. (**c**) Effects of Cl^−^ concentration on SLO-2 channel activity. Shown are representative current traces (holding voltage +30 mV) from SLO-2 (*n* = 11) and SLO-2 + BKIP-1 (*n* = 11), which represent *shk-1(ok1581);bkip-1(zw10)* and *shk-1(ok1581)* strains, respectively. Ca^2+^ concentration in the bath solution was kept constant (100 µM). The segments marked by the dotted rectangles are shown below at an expanded time scale. (**d**) Cl^−^ concentration and SLO-2 open probability (*nP*_*o*_) curves fitted to the Hill’s equation, and comparison of the slope and the half maximal effective concentration (*EC*_*50*_) between the two groups. All values are shown as mean ± SE. The double asterisk (**) indicates a statistically significant difference (*p* < 0.01, unpaired *t*-test).

Given that SLO-2 is activated by Cl^−^ and Ca^2+^ on the cytosolic side^21,29^, BKIP-1 might regulate SLO-2 function through altering its Cl^−^ and/or Ca^2+^ sensitivities. We explored these possibilities by analyzing the effects of BKIP-1 on SLO-2 single-channel activity in inside-out patches of body-wall muscle cells. To identify SLO-2 single-channel events, we obtained membrane patches from mutants of several K^+^ channels expressed in muscle cells using pipettes with a tip resistance of ~20 MΩ, and recorded single-channel activities using a bath solution containing 100 μM Ca^2+^ and 50 mM Cl^−^. Single-channel activities were always observed in patches from *shk-1(ok1581)* but never in patches from either *slo-2(nf101)* or *slo-2(nf101);shk-1(ok1581)* (Fig. 2a), suggesting that SLO-2 is the only contributor to the single-channel activities under our experimental conditions. Because an earlier study reported the observation of SLO-1 single-channel activities in muscle cells under comparable experimental conditions (100 μM Ca^2+^ and 150 mM Cl^−^ in bath solution)^31^, we tried to identify any SLO-1 single-channel activity by using recording pipettes of a much larger tip size (resistance ~2 MΩ). Single-channel events were observed in only a small percentage (~20%) of patches from *slo-2(nf101)* but never in patches from *slo-2(nf101);slo-1(md1745)* (Fig. 2b), suggesting that they resulted from SLO-1. SLO-1 displayed flickery openings with sub-conductance states (Fig. 2b), which is very distinct from SLO-2. Density of SLO-1 in the muscle cell membrane appears to be extremely low; we never had a patch showing more than one active SLO-1 channel even though we spent a lot of time trying to get patches from various areas of the muscle cells, including dense body areas where SLO-1 is enriched^19^.

The dominance of SLO-2 in single-channel events makes it easy to analyze the effect of BKIP-1 on SLO-2. We therefore used pipettes of the smaller tip size in subsequent single-channel recordings. To assess potential effects of BKIP-1 on SLO-2 Cl^−^ sensitivity, bath solutions containing different concentrations of Cl^−^ with a constant concentration of Ca^2+^ (100 μM) were applied sequentially. Cl^−^ increases SLO-2 open probability concentration-dependently in both SLO-2 and SLO-2 + BKIP-1 strains (Fig. 2c,d). BKIP-1 causes a rightward shift of the [Cl^−^] – open probability curve; the Cl^−^ concentration for half-maximal channel activation (*EC*_50_) increased from 34.5 ± 3.8 to 58.1 ± 3.1 mM (Fig. 2d), suggesting that BKIP-1 decreases SLO-2 Cl^−^ sensitivity.

**Figure 3:**
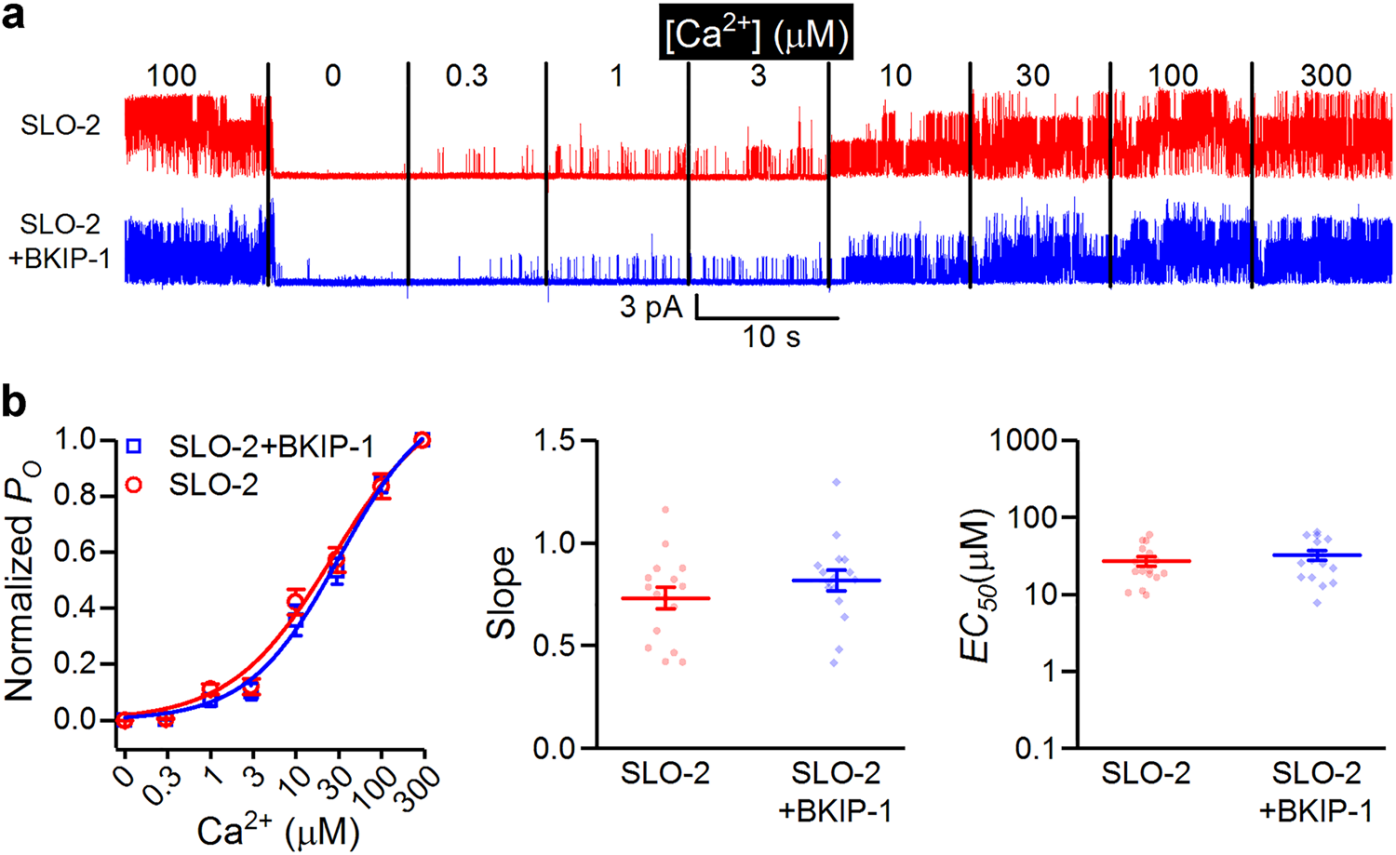
BKIP-1 does not alter SLO-2 Ca^2+^ sensitivity. (**a**) Effects of Ca^2+^ concentration on SLO-2 channel activity. Shown are representative current traces of inside-out patches (holding voltage +30 mV) from body-wall muscle cells of SLO-2 (*n* = 16) and SLO-2+ BKIP-1 (*n* = 16), which represent *shk-1(ok1581);bkip-1(zw10)* and *shk-1(ok1581)* strains, respectively. Cl^−^ concentration in the bath solution was kept constant (50 mM). (**b**) Ca^2+^ concentration and SLO-2 open probability (*nP*_*o*_) curves fitted to the Hill’s equation, and comparison of the slope and the half maximal effective concentration (*EC*_*50*_) between the two groups. All values are shown as mean ± SE.

To assess potential effects of BKIP-1 on SLO-2 Ca^2+^ sensitivity, bath solutions containing different concentrations of Ca^2+^ with a constant concentration of Cl^−^ (50 mM) were applied sequentially. SLO-2 activity increases concentration-dependently when [Ca^2+^] in the bath solution is increased. However, the [Ca^2+^] – open probability curve is indistinguishable between SLO-2 and SLO-2 + BKIP-1 (Fig. 3), suggesting that BKIP-1 does not regulate SLO-2 Ca^2+^ sensitivity.

### BKIP-1 reduces SLO-2 open probability

**Figure 4:**
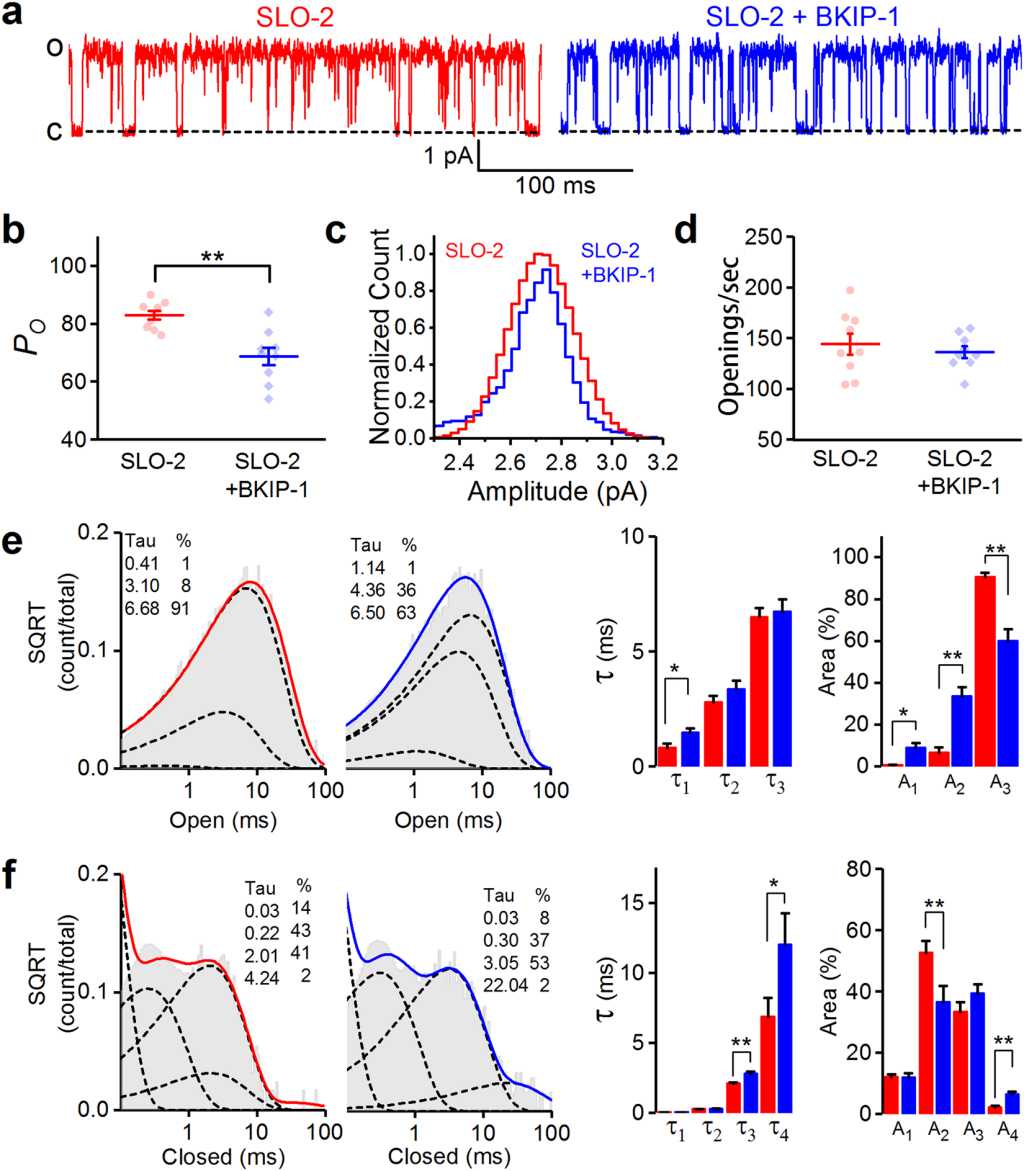
BKIP-1 reduces SLO-2 single-channel open probability (*P*_*o*_) in inside-out patches of body-wall muscle cells. (**a**) Representative current traces from SLO-2 and SLO-2 + BKIP-1, which represent *shk-1*(*ok1581*);*bkip-1*(*zw10*) and *shk-1(ok1581)* strains, respectively. The bath solution contained 50 mM Cl^−^ and 100 µM Ca^2+^, and the holding voltage was +30 mM. (**b**–**d**) Comparison of *P*_*o*_, single-channel amplitude distribution, and opening frequency between SLO-2 (*n* = 9) and SLO-2 + BKIP-1 (*n* = 9). Each dot in (**b** and **d**) represents the mean value of all the opening events of one recording. (**e**) and (**f**) Fitting of open (**e**) and closed (**f**) durations to exponentials, and comparison of τ values and relative areas (%) of the fitted components (indicated by dotted lines) between SLO-2 and SLO-2 + BKIP-1. All values are shown as mean ± SE. The asterisk (*) and double asterisk (**) indicate statistically significant differences at *p* < 0.05 and *p* < 0.01 levels, respectively (unpaired *t*-test).

We selected inside-out patches from body-wall muscle cells with apparently only one channel to analyze the effect of BKIP-1 on SLO-2 single-channel activity. BKIP-1 inhibits SLO-2 open probability (*P*_*o*_) by ~15% without altering either the single-channel current amplitude (SLO-2, 2.8 ± 0.1 pA; SLO-2 + BKIP-1, 2.7 ± 0.1 pA) or open frequency (SLO-2, 144.2 ± 10.5 openings/sec; SLO-2 + BKIP-1, 136.2 ± 5.8 openings/sec) (Fig. 4a–d). Further analyses show that SLO-2 has 3 open states and 4 closed states, and that the inhibitory effect of BKIP-1 on *P*_*o*_ mainly results from a large decrease in the proportion of the longest open state and a big increase in the duration of the longest closed state (Fig. 4e,f). These observations suggest that BKIP-1 likely regulates SLO-2 gating directly.

### BKIP-1 shapes muscle action potentials

**Figure 5:**
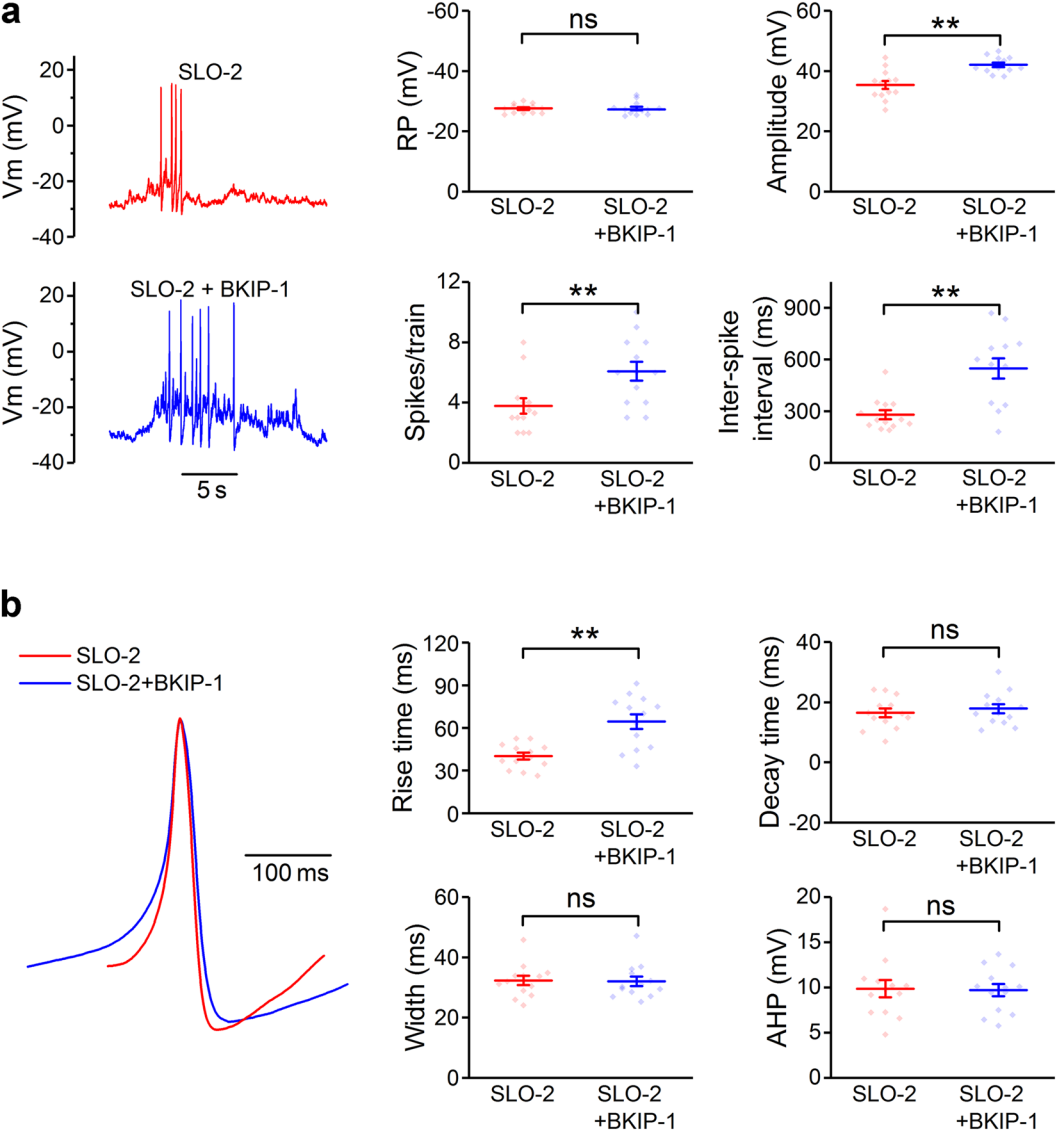
BKIP-1 regulates body-wall muscle action potential properties. (**a**) Sample current-clamp traces from SLO-2 and SLO-2 + BKIP-1, which represent *shk-1(ok1581);bkip-1(zw10)* and *shk-1(ok1581)* strains, respectively, and comparison of the resting membrane potential (RP), the mean amplitude of action potentials (spikes), number of spikes per train, and inter-spike interval. (**b**) Representative action potentials from SLO-2 and SLO-2 + BKIP-1 superimposed, comparison of the 10–90% rise time, 10–90% decay time and mid-peak width of action potentials, and comparison of the amplitude of afterhyperpolarization. Each dot in a graph represents the mean value of all the events of one recording. All values are shown as mean ± SE. The double asterisk (**) indicates a statistically significant difference (*p* < 0.01) whereas “ns” indicates no statistically significant difference (*p* > 0.05) compared between SLO-2 (*n* = 13) and SLO-2 + BKIP-1 (*n* = 13) (unpaired *t*-test).

SLO-2 plays important roles in setting the resting membrane potential and shaping action potentials in *C. elegans* body-wall muscle cells^22^. We therefore determined whether BKIP-1 regulates muscle resting membrane potential and action potentials. BKIP-1 does not affect the resting membrane potential but increases the amplitude of action potentials, the number of spikes per train, and inter-spike intervals (Fig. 5a). In addition, BKIP-1 increases the rise time of action potentials without altering their decay time and mid-peak width (Fig. 5b). BKIP-1 also has no effect on the afterhyperpolarization (Fig. 5b). These effects of BKIP-1 are conceivably due to the regulation of SLO-2 function.

### BKIP-1 inhibits SLO-2 in motor neurons

**Figure 6:**
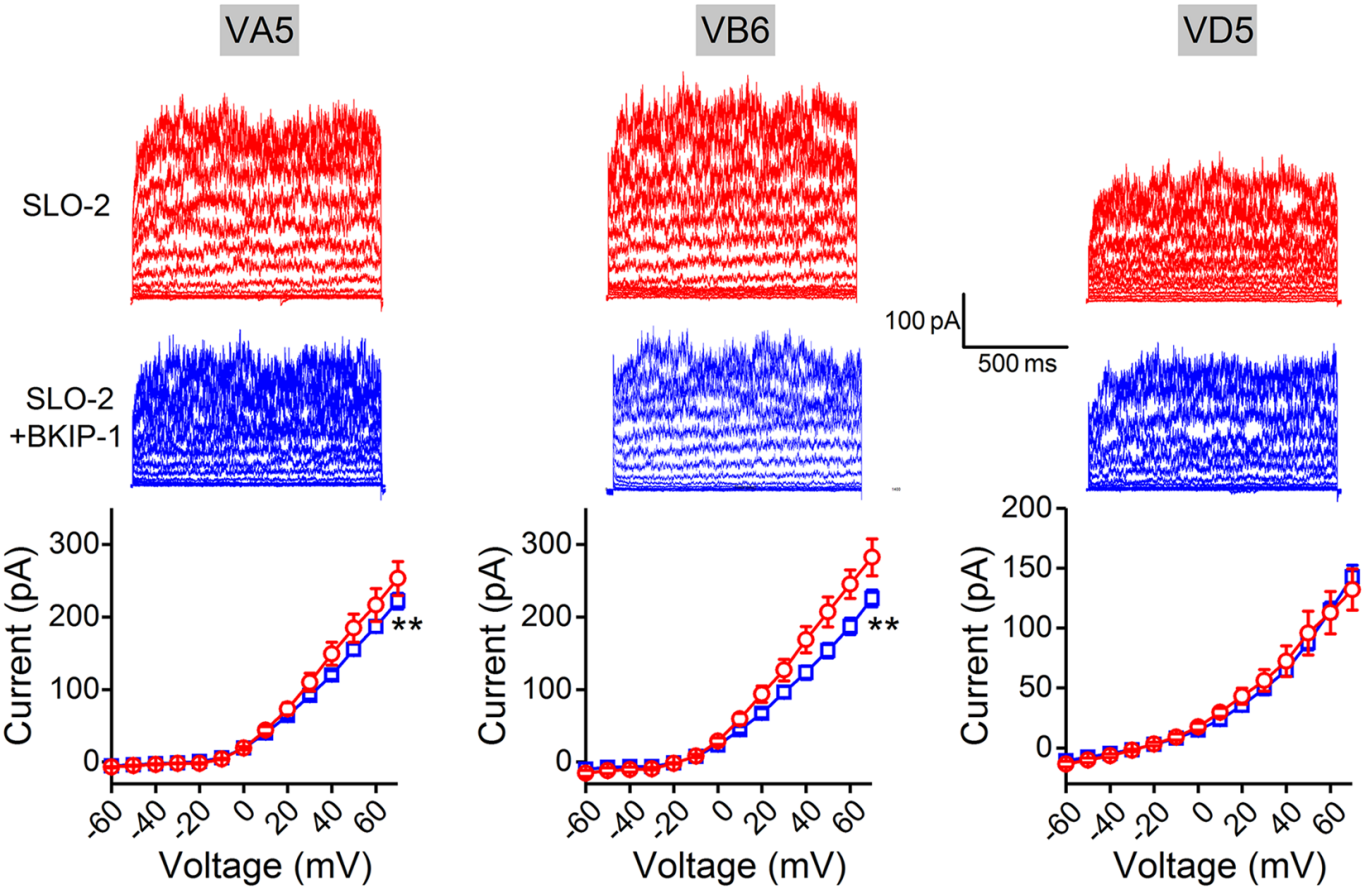
BKIP-1 inhibits SLO-2 whole-cell currents in VA5 and VB6 cholinergic motor neurons but not in VD5 GABAergic motor neuron. Shown are sample current traces and the current-voltage relationship of SLO-2 (VA5, *n* = 5; VB6, *n* = 6; VD5, *n* = 5) and SLO-2 + BKIP-1 (VA5, *n* = 12; VB6, *n* = 12; VD5, *n* = 9), which represent *shk-1*(*ok1581*);*bkip-1*(*zw10*) and *shk-*1(*ok1581*) strains, respectively. All values are shown as mean ± SE. The double asterisk (**) indicates a statistically significant difference at *p* < 0.01 levels (two-way *ANOVA*).

Among motor neurons important to *C. elegans* locomotion are three types: A, B, and D. The A and B types mediate backward and forward movements, respectively, and contract muscles by releasing acetylcholine, whereas the D type relaxes muscle by releasing GABA (γ-aminobutyric acid)^32^. Our previous study showed that SLO-2 is an important conductor of delayed outward currents in VA5, VB6 and VD5, which are representatives of A, B, and D type motor neurons, respectively^21^. To determine whether BKIP-1 regulates SLO-2 in these motor neurons, we compared whole-cell currents between SLO-2 and SLO-2 + BKIP-1. BKIP-1 causes a significant decrease in delayed outward currents of VA5 and VB6 but not VD5 (Fig. 6).

**Figure 7:**
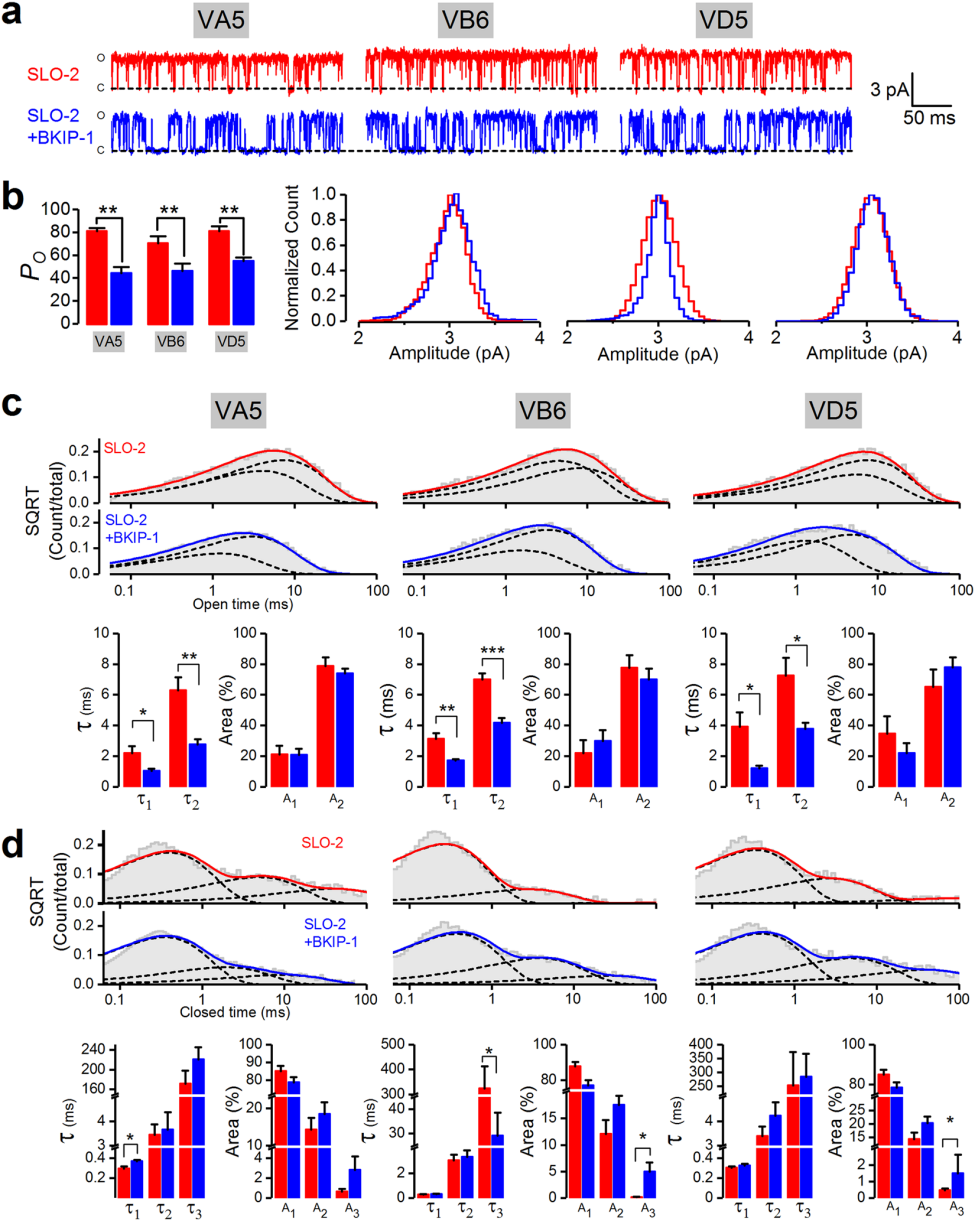
BKIP-1 reduces SLO-2 single-channel open probability (*P*_*o*_) in inside-out patches of representative cholinergic (VA5, VB6) and GABAergic (VD5) motor neurons. (**a**) Representative current traces of SLO-2 and SLO-2 + BKIP-1, which represent *shk-1(ok1581);bkip-1(zw10)* and *shk-1(ok1581)* strains, respectively. (**b**) Comparison of *P*_*o*_ and single-channel amplitude distribution between SLO-2 and SLO-2 + BKIP-1. (**c** and **d**) Fitting of open (**c**) and closed (**d**) durations to exponentials, and comparison of τ values and relative areas of the fitted components (indicated by dotted lines) between SLO-2 and SLO-2 + BKIP-1. *n* = 6 in all groups. All values are shown as mean ± SE. The asterisks indicate statistically significant differences (*p < 0.05, **p < 0.01, ***p < 0.001, unpaired *t*-test).

Because SLO-2 contributes a much smaller proportion of the total delayed outward currents in VD5 (33%) than either VA5 (80%) or VB6 (67%)^21^ and the inhibitory effects of BKIP-1 on delayed outward currents are small even in VA5 and VB6, we wondered whether SLO-2 in VD5 is also regulated by BKIP-1 but this effect was not detected in the recorded whole-cell currents. To address this possibility, we analyzed the effects of BKIP-1 on SLO-2 single-channel properties in patches that apparently contained only one SLO-2 channel. Our analyses show that BKIP-1 inhibited SLO-2 *P*_*o*_ by ~50% in all three neurons without altering the single-channel current amplitude (Fig. 7a,b). Analyses of single-channel open and closed times suggest that there are at least 2 different open states and 3 different closed states, and that the inhibitory effect of BKIP-1 on SLO-2 *P*_o_ mainly results from decreased open durations of the two open states (Fig. 7c,d). Taken together, our analyses of the effects of BKIP-1 on SLO-2 single-channel activity suggest that BKIP-1 inhibits SLO-2 in both cholinergic and GABAergic motor neurons.

### BKIP-1 interacts with SLO-2 carboxyl terminal

**Figure 8:**
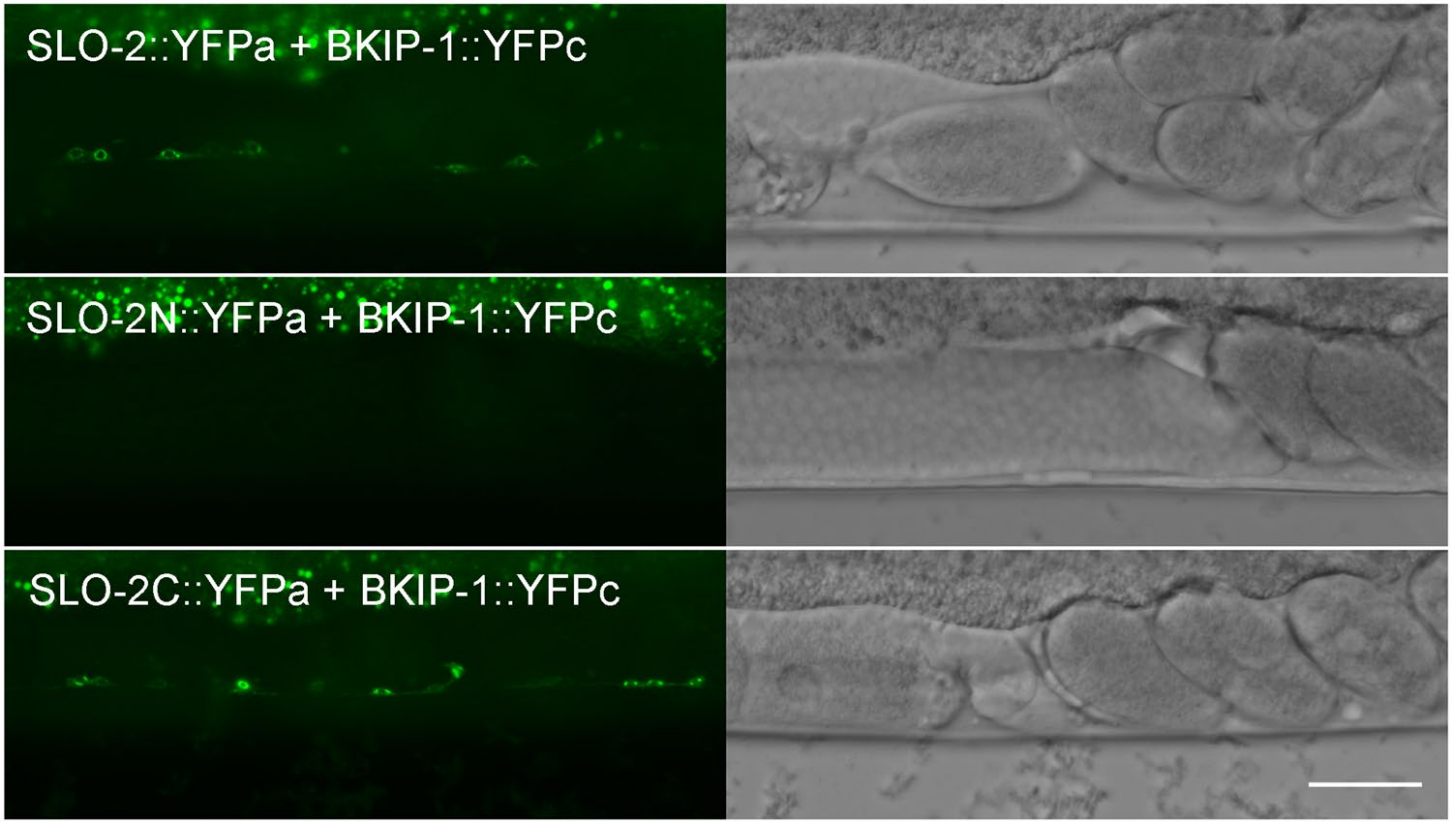
BKIP-1 interacts with SLO-2 in neurons. Bimolecular fluorescence complementation (BiFC) assays were performed by co-expressing SLO-2 and BKIP-1 tagged with the amino and carboxyl terminal portions of YFP (YFPa and YFPc), respectively. YFP signal was detected in motor neurons with full-length SLO-2 and BKIP-1 (*top*). Deletion of SLO-2 carboxyl terminal (*middle*) but not amino terminal (*bottom*) abolished the YFP signal. Shown are representative images of a segment of the ventral nerve cord. The bright signal at the top of each fluorescence image is from auto-fluorescence of the intestine. Scale bar, 20 μm.

We performed bimolecular fluorescent complementation (BiFC) assays^33^ to determine whether BKIP-1 may physically interact with SLO-2 in motor neurons. In these assays, the nonfluorescent amino- and carboxyl-terminal portions of YFP (YFPa and YFPc) were fused to SLO-2 and BKIP-1, respectively. The fusion proteins were co-expressed in neurons under the control of the pan-neuronal *rab-3* promoter (P*rab*-3). An observation of YFP fluorescence would indicate interactions between the two proteins. YFP fluorescence was observed in transgenic animals expressing full-length BKIP-1 (BKIP-1::YFPc) and SLO-2 (SLO-2::YFPa) (Fig. 8 *top*). YFP fluorescence was also observed when YFPa-tagged SLO-2 carboxyl terminal (SLO-2C::YFPa) was used (Fig. 8 *bottom*) but not when YFPa-tagged SLO-2 amino terminal (SLO-2N::YFPa) was used (Fig. 8 *middle*) in the assay. These observations suggest that BKIP-1 interacts with SLO-2 carboxyl terminal.

## Discussion

This study establishes BKIP-1 as a novel auxiliary subunit of SLO-2 through experiments with native cells. Our results suggest that BKIP-1 modulates SLO-2 through several mechanisms, including reducing apparent Cl^−^ and voltage sensitivities, slowing activation rate, and altering either the duration or proportion of open or closed states. These diverse effects of BKIP-1 on SLO-2 are reminiscent of those of mammalian β subunits on Slo1. For example, β subunits may alter Slo1 apparent voltage and Ca^2+^ sensitivities, slow Slo1 activation, and regulate the duration and frequency of Slo1 single channel openings (see references^13,14^ for reviews). The fact that BKIP-1 regulates various biophysical properties of SLO-2 and interacts with SLO-2 carboxyl terminal *in vivo* suggests that BKIP-1 is probably an integral component of SLO-2 channels with important physiological roles.

SLO-2 is expressed in body-wall muscle cells and many neurons, including ventral cord motor neurons^29,34^. In body-wall muscle cells, SLO-2 plays major roles in setting the resting membrane potential, repolarizing action potentials, and producing afterhyperpolarization^22^. In motor neurons, SLO-2 sets the resting membrane potential, and inhibits neurotransmitter release through shortening the duration and reducing charge transfer rate of spontaneous postsynaptic current bursts^21^, which are the electrical signals used by motor neurons to instruct muscle activity^35^. The present study shows that the presence of BKIP-1 causes significant changes in biophysical properties of SLO-2 in both muscle cells and motor neurons, and in muscle action potential properties, suggesting that BKIP-1 is important to the function of SLO-2 in many cells. Although BKIP-1 inhibits SLO-2 *P*_*o*_ in all the cells examined, its effects on channel open and closed states are somewhat variable. *C. elegans* has at least 8 different isoforms of SLO-2 and 2 different isoforms of BKIP-1 due to alternative splicing (www.wormbase.org). Conceivably, the variable effects of BKIP-1 on SLO-2 open and closed states among the muscle cells and neurons could result from the expression of different isoforms of SLO-2 and/or BKIP-1 or the presence of different SLO-2-interacting proteins in the different cells.

BKIP-1 was initially identified as an auxiliary subunit of SLO-1 through a genetic screen for suppressors of a sluggish phenotype caused by expressing a hyperactive SLO-1 in *C*. elegans^24^. SLO-1 is a prominent K^+^ channel in both neurons and body-wall muscle cells^19,20,36^. In neurons, SLO-1 colocalizes with presynaptic markers^20,37^, and serves as a potent negative regulator of neurotransmitter release^19^. In body-wall muscle cells, SLO-1 colocalizes with EGL-19 (Ca_V_1/L-type voltage-gated Ca^2+^ channel)^27^, and plays an important role in regulating Ca^2+^ mobilization^20^. *bkip-1* and *slo-1* mutants are indistinguishable in phenotypes^24^, suggesting that BKIP-1 is indispensable for SLO-1 function *in vivo*. The finding of BKIP-1 as a regulator of both SLO-1 and SLO-2 demonstrates that a single auxiliary subunit may differentially regulate two members of the Slo family within the same cells.

BKIP-1 was first implicated in SLO-2 function in a recent study to determine the roles of SLO-1 and SLO-2 in asymmetric differentiation of a pair of olfactory sensory neurons (AWC) in *C. elegans* ^28^. While single loss-of-function mutants of *slo-1*, *slo-2* and *bkip-1* show normal AWC differentiation, a combination of any two mutants of the three genes disrupts the asymmetry of AWC neurons. These observations led to the suggestion that BKIP-1 is required for the function of both SLO-1 and SLO-2 in AWC neurons. The apparent difference in BKIP-1 effects on SLO-2 between AWC neurons and motor neurons might be due to the usage of different isoforms of SLO-2 and/or BKIP-1 in these cells.

Although SLO-1 and SLO-2 are both expressed in muscle cells and neurons, single-channel events in inside-out patches obtained with the smaller pipette tip size appeared to be entirely due to SLO-2. SLO-1 single-channel events could only be observed in patches of muscle cells using pipettes with a much larger tip size, and even so only ~20% of the patches displayed SLO-1 activity. These observations are very different from those of an earlier study, which reported that single-channel events of SLO-1 are frequently observed and those of SLO-2 run down quickly in inside-out patches of body-wall muscle cells^31^. We do not know whether the apparent differences in results between these two studies are due to differences in experimental conditions or other factors. Because all the experiments of the previous study were performed with worms of wild-type *slo-2* genetic background, it is unclear whether the results or interpretations have been complicated by SLO-2.

The modulatory effects of BKIP-1 on SLO-2 are relatively weak compared with those of many mammalian Slo1 regulatory proteins. For example, the γ1 subunit (LRRC26) and β1 subunit shift the *V*_50_ of Slo1 by over 100 mV^2,11^, and the β2 and β3 subunits cause great inactivation of Slo1^8,10^. Among the possible causes for the relatively weak regulatory effect of BKIP-1 on SLO-2 are a much narrower physiological range of the membrane potential of *C. elegans* muscle cells and neurons compared with that of mammalian neurons and muscle cells. In *C. elegans*, muscle cells have a resting membrane potential of approximately −27 mV, and depolarize to approximately +20 mV at the peak of action potentials^22^; and motor neurons have a resting membrane potential of −46 to −72 mV depending on the types of neurons, and can depolarize to about −20 mV. In contrast, mammalian muscle cells and neurons generally have a resting membrane potential of −70 to −90 mV, and reaches as high as +50 mV at the peak of action potentials. Consistent with this speculation, the effect of BKIP-1 on SLO-1 *V*_50_ is also small (~20 mV). It thus appears that nature has exquisitely tuned the properties of BKIP-1 to allow the proper functions of both SLO-2 and SLO-1 *in vivo*.

Slo1 and Slo2 channels are abundantly expressed in various mammalian tissues, with many cell types potentially expressing both of them. The regulation of two different Slo channels by one auxiliary/regulatory protein is probably not a phenomenon unique to worms but may occur in mammalian systems as well. This kind of regulations has the potential to fine-tune channel biophysical properties to specific cellular needs under physiological conditions.

## Methods

### *C. elegans* culture and strains

Worms were raised on NGM plates with a layer of OP50 *E. coli* at 22 °C inside an environmental chamber. The following strains were used in this study: Wild type (Bristol N2). RB1392: *shk-1*(*ok1581*). LY101: *slo-2*(*nf101*). ZW477: *bkip-1*(*zw10*). ZW902: *slo-2(nf101);bkip-1*(*zw10*). ZW903: *shk-1*(*ok1581*);*slo-2*(*nf101*). ZW904: *shk-1(ok1581);bkip-1*(*zw10*). ZW1150: *shk-1(ok1581);bkip-1*(*zw17*). ZW1156: *zwEx2*50*[Prab-3::slo-2::YFPa*(*wp1783*), *Pmyo-2::mStrawberry(wp1613)]; zwEx249[Prab-3::bkip-1::YFPc(wp813), lin-15*(+)]; *lin-15*(*n765*). ZW1157: *zwEx251[Prab-3::slo-2N::YFPa(wp1784), Pmyo-2::mStrawberry(wp1613)]; zwEx249*[*Prab-3::bkip-1::YFPc(wp813), lin-15*(+)]; *lin-15(n765)*. ZW1158: *zwEx252[Prab-3::slo-2C::YFPa(wp1785), Pmyo-2::mStrawberry(wp1613*)]; *zwEx249[Prab-3::bkip-1::YFPc(wp813), lin-15*(+)]; *lin-15*(*n765*).

### Bimolecular fluorescence complementation (BiFC) assay

BiFC assays were performed by co-expressing in neurons SLO-2 and BKIP-1 tagged with the amino and carboxyl terminal portions of YFP (YFPa and YFPc), respectively. The cDNAs encoding the full-length, the amino terminal portion (1-327aa), and the carboxyl terminal portion (328–1107aa) of SLO-2 were independently cloned into a plasmid containing P*rab-3* and YFPa DNA sequence. *bkip-1* cDNA was cloned into a plasmid containing P*rab-3* and YFPc DNA sequence. The P*rab-3::bkip-1::YFPc* plasmid (wp813) was first injected into the *lin-15(n765)* strain to establish stable transgenic lines. The transgenic worms from a representative line were then injected separately with the three different *slo-2* plasmids: P*rab-3::slo-2::YFPa* (wp1783), P*rab-3::slo-2N::YFPa* (wp1784), and P*rab-3::slo-2C::YFPa* (wp1785). A P*myo-2::mStrawberry* plasmid (wp1613) was co-injected to serve as a transformation marker. Images of transgenic worms were taken with a digital ORCA-Flash4.0 CMOS camera (C11440-22CU, Hamamatsu Photonics, Japan) mounted on a Nikon TE2000-U inverted microscope equipped with EGFP/FITC and mCherry/Texas Red filter sets (49002 and 49008, Chroma Technology Corporation, Rockingham, VT, USA).

### Electrophysiology

Electrophysiological experiments were performed with adult hermaphrodites. An animal was immobilized on a glass coverslip by applying Vetbond^TM^ Tissue Adhesive (3 M Company, St. Paul, MN). Application of the glue was generally restricted to the dorsal anterior portion of the animal, allowing the tail to sway freely during the experiment. A short longitudinal incision was made along the glued region. After clearing the viscera by suction through a glass pipette, the cuticle flap was folded back and glued to the coverslip, exposing several ventral body-wall muscle cells and a small number of motor neurons anterior to the vulva. The dissected worm preparation was treated with collagenase A (Roche Applied Science, catalogue number 10103578001, 0.5 mg/ml) for 10–15 sec and perfused with the extracellular solution for 5 to 10-fold of bath volume. Borosilicate glass pipettes were used as electrodes for voltage-clamp whole-cell or single-channel recordings. In whole-cell recordings, pipette tip resistance for recording from body-wall muscle cells was 3–5 MΩ whereas that for recording from motor neurons was ~20 MΩ. Classical whole-cell configuration was obtained by applying a negative pressure to the recording pipette. Series resistance was compensated to ~70%. In single-channel recordings with inside-out patches, pipette tip resistance was 20–30 MΩ for both muscle cells and motor neurons. The specific motor neurons VA5, VB6 and VD5 were identified based on their anatomical locations, as described earlier^21^. Voltage-clamp experiments were performed with an amplifier (Multiclamp 700 A, Molecular Devices, Sunnyvale, CA, USA), a digitizer (Digidata 1550B, Molecular Devices), and the Clampex software (version 10, Molecular Devices). Data were filtered at 2 kHz and sampled at 10 kHz.

### Solutions

In the whole-cell voltage- and current-clamp experiments, identical extracellular and pipette solutions were used. The extracellular solution contained (in mM) 140 NaCl, 5 KCl, 5 CaCl_2_, 5 MgCl_2_, 11 dextrose and 5 HEPES (pH7.2). The pipette solution contained (in mM) 70 KCl, 20 KOH, 50 K^+^ gluconate, 8 HCl, 5 Tris, 0.25 Ca^2+^ gluconate, 1 MgCl_2_, 10 sucrose, 5 EGTA and 5 Na_2_ATP (pH 7.2). In the experiments with inside-out patches, the pipette solution contained (in mM) 150 K^+^ gluconate, 1 Mg^2+^ gluconate and 10 HEPES (pH 7.2) whereas the bath solution varied depending on specific experiments. The bath solution used to determine the effects of Cl^−^ on SLO-2 activity contained (in mM) 1 Mg^2+^ gluconate, 0.1 Ca^2+^ gluconate, 5 HEPES, and various concentrations (total 150 mM) of KCl (0, 5, 10, 20, 40, 80, 120 mM) and K^+^ gluconate (adjusted based on the concentration of KCl) (pH 7.2). The bath solution used to determine the effects of Ca^2+^ on SLO-2 activity contained (in mM) 100 K^+^ gluconate, 50 KCl, 5 HEPES, and various concentrations of Ca^2+^ gluconate and Ca^2+^ buffer (pH 7.2). Specific concentrations of free Ca^2+^ were achieved by adding 5 mM EGTA (no Ca^2+^ addition) for 0 free Ca^2+^, 3.23 mM Ca^2+^ gluconate plus 5 mM EGTA for 0.3 μM free Ca^2+^, 4.3 mM Ca^2+^ gluconate plus 5 mM EGTA for 1 μM free Ca^2+^, 1.6 mM Ca^2+^ gluconate plus 5 mM HEDTA for 3 μM free Ca^2+^, 3.07 mM Ca^2+^ gluconate plus 5 mM HEDTA for 10 μM, and 0.03, 0.1, and 0.3 mM Ca^2+^ gluconate for 30 μM, 100 μM and 300 μM free Ca^2+^, respectively. Free [Ca^2+^] was calculated using online software (http://web.stanford.edu/~cpatton/webmaxcS.htm). The 100-μM Ca^2+^ solution was also used in experiments analyzing SLO-2 single-channel biophysical properties. In the experiments analyzing the effects of Cl^−^ or Ca^2+^ on SLO-2 activity, the recording pipette was inserted into the opening of a Perfusion Pencil^TM^ (Automate Scientific, Inc., Berkeley, CA, USA) through which various solutions were perfused using a perfusion controller (VALVELINK8.2, Automate Scientific, Inc.).

### Data analyses

Clampfit (Molecular Devices) was used for the quantification of most electrophysiological data. The amplitude of SLO-2 or SHK-1 whole-cell current was quantified from the mean amplitude during the last 100 ms of each voltage step. The SLO-2 whole-cell current data were then converted to conductance (*G*) and fitted to the Boltzmann function *G*/*G*_*max*_ = 1/{1 + exp[(*V*_50_ − *V*)/*k*]}, where *G*_*max*_ is the fitted value for maximal conductance, *V*_50_ is the voltage of half maximal activation of conductance, and *k* is the term for the voltage dependence of activation in units of mV. The activation rate of SLO-2 whole-cell current was quantified by fitting the initial 500-ms current trace of each voltage step to two exponentials. The open probability SLO-2 in patches used to determine the effects of varying Cl^−^ or Ca^2+^ concentration was quantified from the current integral above the baseline, and the concentration-open probability curves were fitted with the equations *y* = *Bottom* + (*Top*-*Bottom*)/(1 + 10^((EC50-*X*)*slope) (for Cl^−^ response) and *y* = *Bottom* + (*Top*-*Bottom*)/(1 + 10^((Log*EC*50-*X*)*Slope)) (for Ca^2+^ response), where *Top* and *Bottom* are the maximum and minimum responses, *X* is the test concentration, and *Slope* is the Hill slope. Patches containing only one channel were used for quantifying SLO-2 opening frequency, single-channel current amplitude distribution, and open and closed time analyses. The QuB software (https://qub.mandelics.com/) was used to fit open and closed times to exponentials, and to quantify the τ values and relative areas of the fitted components, which were automatically determined by the software. The entire recording of each experiment (30 sec duration) was used in such analyses. Statistical comparisons were performed with OriginPro (version 9, OriginLab, Northampton, MA, USA) using either unpaired *t*-test or one-way analysis of variance (ANOVA), as specified in figure legends. All values are shown as mean ± s.e. *p* < 0.05 is considered to be statistically significant. The sample size (*n*) equals to either the number of cells (Figs 1, 5 and 6) or membrane patches (Figs 2, 3, 4 and 7). Data graphs were made with OriginPro.

### Data availability

All data generated or analyzed in this study are included in this published article.
